# Proceedings of the Maryland and District of Columbia Dental Association. Annual Meeting

**Published:** 1881-01

**Authors:** 


AMERICAN JOURNAL

OF

DENTAL SCIENCE.

Vol. XIV. THIRD SERIES—JANUARY, 1881. No. 9.

### ARTICLE I.

*Proceedings of Maryland and District of Columbia Dental Association.—Annual Meeting.*

WASHINGTON, OCTOBER 27, 1880.

The Association was called to order by the President, Dr. M. W. Foster, at 11 A. M., and was opened with prayer by the Rev. John Lanahan.

Dr. Donaldson, Chairman of the Executive Committee, stated that by an oversight no one had been appointed to deliver the welcoming address. He therefore, as chairman, extended to those from abroad a cordial welcome to the meeting and the city, and expressed the hope that this would be an interesting and useful meeting.

The President, Dr. Foster, then delivered the following address:

*Gentlemen and Fellow Members of the Maryland and District of Columbia Dental Association:*

In looking over the addresses of my predecessors I have found in them so much that is useful and instructive, I fain would in lieu of this address, present them to you again, if allowable, entire.

I shall quote from them, or other addresses, whenever I find the language more appropriate or the idea better conveyed than my own. We are again reassembled for the purpose of interchanging ideas; to brighten ourselves by attrition to new facts; to discuss and treat *new views* of old facts; to correct our past errors; to combat prejudice; to dessentinate useful knowledge and for social and friendly converse.

We have systematized our labors by the formation of the several sections, that it only remains for us to hear their reports and discussions on the same. It is therefore, my pleasant duty to call your attention to the necessity of a careful collection of statistics, for by these we stand or fall.

If our Association is to be a power in the land (as I hope and trust it may be) it must pay strict attention to details; must so watch its actions and be so jealous of its verdicts, as to elicit admiration and challenge criticism. To be able to accomplish this successfully we must throw proper safeguards around ourselves, and our membership, so we may say with the Apostle of the Gentiles "We are many, yet but one body. If one member suffers, all members suffer with it, or if one member be honored, all the members rejoice with it."

The great secret of success in life is sterling integrity; the great secret in all successful associations, is confidence: Can you have the one without the other? If then we expect the public, who is looking to us for correct information in our specialty, to believe in our edicts, we must first have confidence in ourselves; to attain this I would suggest to the committee in charge, a careful supervision of future membership, as one of the means, in my judgment, to place us in a proper position before the public.

Among the many advantages derived from the Association, is the opportunity furnished to the student to become better acquainted with the profession in all its different phases; it forms a guard of safety to the honest and competent practitioner, and a scourge to the charlatan; it will supply the public with the means of judging between scientific competence and ignorant practice; and thus save it from injury and imposition.

An Association to be in the best possible condition for progression, must be free, must throw to the winds all old-fogy ideas, be ready and willing to receive new light, and yet strong enough to hold fast to all the best of past experience; or as Dr. Donaldson said in his address, "to be *properly careful* in taking hold of new modes of practice."

Without wishing to be personal, or too greatly credit this association with *all* the wisdom extant, I may safely say without fear of successful contradiction, that for its numbers, it is as Dr. Coy said, the "biggest kind of a little association;" and from the number of known level headed gentlemen present, one can feel assured that anything of merit will receive from it proper consideration and commendation, and any false theory will as emphatically receive its disapprobation and censure.

The many advantages this Association possesses over others, is due in a great measure to its local surroundings; having at all times easy access to the Smithsonian and other institutions of like character, and the co-operation of Army and Naval surgeons (who were furnished some three years since with carefully prepared circulars asking them to note the climatic and food effect on the teeth of the different nationalities where they might be stationed); these are advantages, I claim, not possessed by kindred associations, and, originating with this, is cause for a reasonable degree of pride and congratulation.

And in this connection, I would urge upon the sections the necessity of careful revision of their reports, so that nothing may go from here with the approval of the Association, that is not entirely reliable and authentic. For instance, when two practitioners of equal eminence in the profession make statements opposite in the extreme of the action of a remedy, or remedies; would it not be well to announce authoritatively through the section in charge, whether one remedy similarly applied can kill or cure at the same time?

There is one other important point which I think, clearly comes within the province of this Association, and I would ask the careful consideration of members to this subject. What influence does it exert on our institutions of learning? Have we not the right to a respectful hearing and consideration of any authoritative paper emanating from this or kindred Associations? If so, would it not be well and proper to have what might be termed a supervisory committee elected, whose duty it would be to suggest any needed reform for the future, or ask the correction of a present defect.

Associations are but foster mothers to Dental Colleges, from them they receive support through students of its members; is it not reasonable then, to assume that, Associations are in a better position to offer council to the Colleges than any other body?—not excepting the regents or trustees of said institutions. The Associations are as deeply interested in dental education, and not being connected with any institution are without the influence, however unconsciously it may be exerted, of the Faculties.

The American Dental Association through its Committee on Dental Education made the following report, August 5 th 1879. (1.) "That all dental schools shall demand some degree of proficiency in English education as preliminary to entering upon professional study. (2.) Not important to notice at this time. (3.) That this association claims the right to exercise a general supervision over the whole subject of dental education."

It is becoming each year more apparent and partially accepted by a number of the Colleges, that interested parties pecuniarily, are not the proper ones to grant diplomas. Dr. Elisha Towusend, in his address before the Pennsylvania Society of Dental Surgeons, said "Diplomas do not confer perfection; and sharp eyed quackery and earnest mediocrity, will occasionally teach the best how much they need to learn, as well as to show them how much they ought to teach."

The more intelligent and advanced men in our profession endorse the above and partial action of the Colleges as an advance in the right direction ; the lesser number combat the action as harmful to the colleges at *present*, tacitly admitting it to be wise and proper legislation for the future.

Believing as I do that associations have the right to a proper supervision over the colleges of this country ; I would suggest some action be taken by this meeting upon the subject, having perfect confidence that whatever action may be taken, would be just and result in mutual benefit.

I can more clearly present my views by quoting Dr. Hunt, who said, "I fully agree that there should be a single association in this country which should have the legitimate, conceded and delegated right to control colleges as to their general standing, and as to their standard of education."

Do colleges need this supervision, or is it presumption on the part of the Association to offer suggestions?

When one of our leading Universities in its annual circular makes the statement "a preliminary examination will be necessary commencing with the regular course October, 1st 1881," the closing lines are thus italicised ; "*Students matriculating before the* 1*st of October will not be required to undergo the preliminary examination.*"

The question naturally arises: What constitutes the difference in a students attainments between the 30th of Sept., and the 1st of October. It is certainly confusing to "lookers on in Vienna."

The following couplet I think appropriate, and might go with the announcement:

"Mr. Orator Puff had two tones to his voice,
 The one squealing thus, and the other down so ;
In each sentence he uttered he gave you your choice,
 For one half was B alt, and the other G low,
  Oh! Oh! Orator Puff,
 One voice for an orator is surely enough.

But he still talked away, spite of laughs and of frowns,
So distracted all ears, with his ups and his downs,
That a wag once, on hearing the orator say
"My voice is for war," cried which of them, pray?

It is my duty to call your attention to the failure of the bill "for the better protection of dentistry," presented to our last legislature; the failure of its passage was not due to any lack of interest or labor on the part of your committee, but from captious opposition outside; the bill could have been passed in an amended form, but the acceptance of this would have nullified its most important features; your committee thought best to withdraw it.

I now suggest that this meeting pass a resolution calling a mass meeting of all dentists in the State of Maryland to consider and present in force, a bill for the better protection of dentistry in our State. We surely cannot afford to be so far behind our sister States.

Gentlemen, for your kindness in honoring me with the position of your executive officer for the past year, receive my heartiest thanks. I now throw myself on your forbearance and ask your assistance in any imperfect ruling, trusting you will credit the error to the head and not the heart.

Dr. Hunt said it was usual to have a committee appointed to make a report upon the President's address, and moved that a committee of three be appointed for this purpose. The President appointed Drs. Hunt, Thompson and Coyle.

Dr. Donaldson moved that the reading of the proceedings be dispensed with. The motion was carried.

Dr. Winder made a verbal report in regard to the action taken by the Legislature of Maryland in regard to the Dental Law, stating that the bill had failed to pass.

Dr Noble moved the report be accepted and the committee be discharged. Carried.

Dr. Donaldson, chairman of the Executive Committee, reported the following hours for meeting, which report was accepted:

For the morning session from 11 until 1 o'clock; for the afternoon session, from 1½ until 4½ o'clock, and the evening session from 7 until adjournment.

He suggested that the evening meeting of Thursday be held at 8 o'clock on account of a meeting of the board of visitors of the Baltimore Dental College, which would take some of the members away. The President said it would be so ordered.

The committee also reported that there would be clinics Thursday morning at nine o'clock.

Dr. Hunt moved that the Executive Committee take into consideration and report upon a time for holding the annual meetings in the future that would not interfere to such an extent with the engagements of the members. The motion was carried.

Dr. Hunt, chairman of Scholarship Committee, reported the name of Benj. Flannigain, of Baltimore, for the scholarship in the Baltimore College of Dental Surgery. The report was adopted and Mr. Flannigain selected.

Reading of papers being in order, the first section was passed for the present on account of the absence of a paper. Dr. Hunt apologised for not having a paper on Metallurgy and Chemistry of Metals, saying he had been very busy and had not prepared one.

On motion of Dr. Waters the meeting adjourned until 1½ o'clock.

---

### Afternoon Session.

There was no business transacted at the afternoon session on account of the absence of many members with important papers.

---

### Evening Session.

At the evening meeting Dr. M. H. Webb read the following paper:—

*Restoration of Contour the only Way to Keep Permanently Separate the Margins of Enamel of the Proximate Surfaces of the Teeth, and Prevent Recurrence of Decay.*

Restoration of the contour of a part or of parts of the crown of a tooth, signifies not only the building out and finishing of the gold to the line that defined the figure originally, but by it is meant the knuckling-ups of the tooth restored to the one adjoining in such a manner that the margins of enamel may be free from contact and that food cannot be forced between the teeth from the masticating surface.

For the restoration of contour, it is necessary to gain space to prepare the cavity, pack the gold, and finish the filling. This must be done either by wedging at the time of performing the operation or by previously pressing wood, linen, tape, or cotton between the teeth. After sufficient space has been made in this manner, white gutta-percha should be placed in the cavity and between the teeth, and remain a day or two or till the irritation incited by the wedging has passed away. Teeth ought not to be separated by rubber because of the irritation which it induces. It is often best to place a wedge of orange wood between the teeth, even after having pressed them apart, not only to gain more space than that had from previous wedging, but to stay the tendency to their approaching each other, and to steady the organs for the performance of the operation, particularly if the irritation from the slow wedging has not passed away so as to admit of the insertion of all or almost all, the gold by aid of the mallet. Space enough to insert and finish fillings, may be gained with less difficulty, by the rapid wedging of the incisor than of the bicuspid and molar teeth, especially when the cavity extends almost to the margin of cementum, or the teeth are firmly set in the alveolar process; however, in these and other cases, the gold should be built into and from the starting point, along the cervical wall and to the part where the convexity of the filling must be gradually increased, and when this much of the gold has been inserted by aid of the mallet and smoothly finished with fine separating or other files, a wedge of orange or other hard wood ought to be inserted between such gold and the tooth adjoining and sufficient space thus secured to enable the operator to so complete the operation that there shall be no space between the teeth, except at and near the necks. Unless separation by pressure is made in some such manner as indicated, gold cannot be so finshed as to have the teeth come closely together and prevent the wedging of food between them, except, perhaps, in the case of a young person when the space left from finishing the fillings with fine files and emery cloth or paper may again close. Pressure ought not to be too long continued or rudely made else circulation in the capillaries and protoplasmic bodies of the pericementum may be so interfered with as to prevent return to the normal condition of the parts after the wedge has been removed.

After sufficient space has been gained, the rubber dam should be applied to the two teeth separated by pressure and the tooth adjoining each that those pressed apart may not come together during the performance of the operation or insertion and finishing of the fillings, and that the organs may be the better operated upon. A cavity within the proximate wall of a bicuspid or molar tooth should be opened into from the masticating surface, excepting in rare cases, because even when but little tissue appears to be disintegrated, this surface would be almost reached upon the perfect removal of the decalcified tissue and the plate of enamel be liable to fracture if hard substances were to come in contact with it during mastication of food. It is far better to cut away the enamel between the cavity and masticating surface than to remove tissue from the entire proximate wall; besides, a clear view is thus had and the whole operation can be performed in a more satisfactory manner. The sulci and fissures are usually imperfect, and should be prepared and filled in connection with, and at the time of, the performance of the operation within the proximate wall. That operations may be successful, each and every cavity must be so prepared that no disintegrated tissue remains, except a little discolored dentine just over the pulps, which should be left for its protection. The margins ought to be evenly and smoothly finished with fine, sharp burs files and emery-cloth, (the edges may be beveled in some cases but not rounded), and a groove should be cut in the dentine along each wall of the cavity. This groove should be about a sixty-fourth of an inch deep and must be made in the dentine just within and near the line of both the buccal and palatal or lingual portions of enamel and ought to extend from the masticating surface to the cervical wall, which should not be cut in that manner (excepting in some cavities in the incisor teeth,) because of there not being such a body of tissue at that part as to make it safe to remove any of it for the purpose named. In the preparation of a cavity in the proximate wall of the enamel of a bicuspid or molar tooth, enough of the tissue near both the buccal and palatal or lingual walls must be cut away, to so restore the contour of the parts and finish the fillings, as to keep the margin of enamel free from contact with the tooth adjoining. This is the only way to keep permanently separate the margins of enamel of the proximate surfaces of the teeth, and prevent recurrence of decay.

After the preparation of most cavities, particularly those that are deep, carbolic acid ought to be applied, to serve not only as a disinfectant, but to coagulate the protoplasm of the ends of the fibres in the dentinal canaliculi and partially obtund sensation. In those cases where thermal changes may produce more than the usual shock in healthy tissue after the gold has been inserted or the operation completed, oxy-chloride of zinc ought to be placed in the bottom of the cavity, as a non-conductor of the currents incited by heat and cold in the dentinal fibers.

When a cavity within the proximate wall of a bicuspid or molar tooth has been prepared as described, a startingpoint should be made in some part of the cervical wall, and in that portion of the dentine which shall be the safest, along the boundary between it and the enamel or cementum. This point, in which to start the filling, should only be deep enough to retain the small pieces of gold first introduced, while other pieces are being built upon them and the filling carried along to the groove in each wall of the cavity. The gold ought to be built against every portion of the dentine, packed as nearly perfect as possible along the enamel and a little beyond its margins, and carried fully to the line that originally defined the contour of the part. To thus perform an operation it is necessary to build that part of the gold nearest the buccal and masticating surfaces, against the proximate surface of the adjoining tooth. By performing this operation with the electro-magnetic mallet, the passing of the packing instrument from off the edge against or over which the gold is being placed, may not only be avoided, but the surplus material can be so trimmed away during the performance of the operation, that comparatively little trimming is afterwards necessary.

Cohesive gold foil, No. 30 or 60 for large, or foil folded to No. 16 or 20 for small fillings, and not touched with the fingers, ought to be used in the performance of all operations, and should be made compact by the use of the electro-magnetic mallet, when it is carefully operated, especially in the cases just referred to, and those where frail edges of enamel are to be supported and protected. All gold should be cohesive, particularly where a mallet is used, so that each piece may remain just where it is placed, and also that the filling may always be one and inseparable. When foil has been so impacked that the substitute for lost tissue is complete, a fine saw or file should be used, to cut away the surplus material to the prepared edge of enamel against which the gold is placed, and to aid in shaping the filling like the original contour of the part; after which narrow strips (a line or an eighth of an inch wide) cut from fine emery-cloth or paper should be so manipulated as to properly finish the surface of the gold. When this has been done and the rubber dam removed, the finishing should be completed by the use of fine pumice and silex on linen tape. The gold at the masticating surface should be trimmed down with fine burs and made concave (or contour in such cases also); that is, finished down to represent the original figure or the outline of the part operated upon. The gold should be placed in the cavity as to be flush with the prepared margins of the enamel and made concave when such concavity is indicated. Fine burs should be used for the purpose of trimming and shaping such fillings, and also the palatal surface of the gold in the incisor and cuspid teeth, because the form of the remaining part or parts of the cusps, and prepared edges of enamel against which the gold is placed, may be changed or disfigured and the tooth be made less useful when corrundum cones are used. The polishing of the surface referred to may be done with pumice and silex mounted upon suitably shaped points of wood, leather or rubber. In each and every case the gold ought to be built out to the original contour of the part and a little beyond the margin, then finished down to the surface of the enamel so that the outline of the whole filling shall be the same as the line that bound, defined or terminated the lost tissue. This line ought to be fully restored, particularly the proximate surfaces of the bicuspids and molars, and the gold should be carefully trimmed down to, and finely finished with, the margin of enamel, which margin, in these cases more particularly than in others, must be free to prevent recurrence of decay. If gold be not placed compactly against, and be not flush with the edges of enamel, the operation is not such as is demanded for the preservation of the remaining tissue. A plane surface of gold should not be made, because the tooth thus operated upon and the one adjoining may move closely together, and disintegration of enamel then take place near the part in contact.

Case after case could be cited where disintegration has taken place near the buccal or palatal wall of the enamel, that came in contact along the proximate surface against which gold (and other material as well) had been placed, while other parts around the same filling remained free from decay. Recurrence of decay did not take place in the same mouth where there was such full restoration of the contour of missing tissue and close knuckleing-up of the teeth as to leave the margins of enamel free from contact and prevent the wedging of food between the proximate surfaces thus restored.* Restoration of contour prevents contact of the margins of enamel, and this prevention is necessary, especially when the tissues of the organ operated upon, are not fully calcified. The contour of missing tissue ought always to be restored with gold, that the enamel of one organ may be free from contact with that of the next in the arch, and also that a part of the gold in one, may be in close contact with the normal tissue of, or a filling (if one has been inserted) in the adjoining tooth, when disintegration is likely to take place, because of the freedom from proximity and the cleansing (by the saliva or fluids taken into the mouth if by nothing else) of the margins of enamel against which the gold has been placed.

All operations ought to be performed as artistically and made as nearly perfect as possible, so that if gold is exposed to view, its appearance shall be beautiful and rather pleasing than otherwise. When operations have been so performed as to entirely prevent fluids or semi-solids from entering between gold and the tissue against which it is placed, and all discoloration has been removed from the surface of remaining tissue, the gold-tint may be seen through the light walls or edges of translucent enamel soon after the removal of the rubber dam and completion of the operation. If an opaque or dark line or spot be

* For the instruction to restore so fully as I now do the contour of the teeth, and "knuckle" them up closely to each other (as well as for instructions in other directions,) I am indebted to Dr. W. H. Atkinson.

visible at or near the parts where gold ought to be in contact with the dentine or enamel, the operation has been imperfectly performed, and if not re-performed, as it ought to be, chemical action may soon follow and the entire filling prove a failure. If operators were always very thorough in their work, there would be no occasion to refer the cause of a failure in dental operations to chemical action, or to the incompatibility of filling material with dentine.

Disintegration commences just above or near the part of enamel in contact in the upper, and just below it in the lower, and soon takes place along the entire proximate surface, and extends to the dentine in which tissue decay makes rapid progress, but in those cases where the system is in good condition, and the tissues are first-class, disintegration takes place slowly and is not so extensive, so that where decalcification of enamel is only superficial it can be removed, and, after a finely finished surface is made, decay will not be likely to recur. In no case should a permanent space be made or left between the teeth, for even in such cases as those just mentioned food may so wedge against the gum as to bring about recession of this tissue and exposure of the necks of the teeth, and finally lead to the formation of a cavity of decay at the part referred to. Permanent separations ought not to be made for the reasons that they interfere with mastication, annoy the patient, and, with few exceptions, do not prevent disintegration upon or about the surfaces that have been cut. The teeth again come in contact almost invariably, excepting where antagonists prevent them; food wedging between them undergoes fermentation and disintegration takes place, and that too, in a part of the tooth where it is difficult to perform a first-class operation. This may not take place, however, till long continued pressure of food paralyzes the nerves in the papillæ throughout the gum-tissue pressed upon, and breaks the circuit or obstructs the movement of the molecules of living matter through that fine, reticulated line between the gum (and all other tissue) and the brain, and the patient is therefore not notified of the presence of such obstruction to the neural and vascular circulation. This condition of the tissue as inevitably leads to its return to the embryonal state as does interference with the nutrition of any other part of the system.

In those cases where tonicity is at normal standard the gum is so close to the necks of the teeth as to prevent the lodgment of foreign matter beneath the margins; not only this but the tissue fills the space between the teeth almost entirely and protects the parts it covers. The gum should therefore be protected by full restoration of the contour of the enamel that is missing, and the gold ought to be finely finished at all points, that there may be no obstruction to the tissue again closing around the neck of the tooth operated upon. In this manner the margins of enamel at or near the neck of the tooth against which the gold is placed and smoothly finished, is protected by the gum, and, if the whole operation has been properly performed, recurrence of decay at that part is prevented. If disintegration does not extend to, or beneath the margin of the gum, both the enamel and dentine of the proximate, as well as buccal surfaces of the teeth, (especially those in which calcification of enamel is imperfect) ought to be cut away with fine burs, to about the sixteenth of an inch above the part where the gum closes around the tooth, so that when the operation is completed this part may be protected from foreign matter. When the necks of the teeth are kept separate as in nature, and the gum is in normal condition, it protects the portion of enamel, and well inserted and finely finished gold which may be beneath its margins so perfectly that disintegration, and even discoloration, is prevented. In after years there may be lack of nutrition to the gum, or the circulation may not be full or free in the capillaries of the part, and the tissue may not close around the necks of the teeth so tightly, but become less firm in texture or commence to return to its embryonal condition. If there be such an acid condition as to disintegrate the enamel, decay may then take place above or about the cervical margin of the filling, but if this be not the case and the saliva be alkaline, deposit of calcareous matter may take place beneath the margin of the gum, and, though there be no recurrence of decay, pericementitis may follow and deposition of such matter prevent the rebuilding of the tissue or keep irritated, or finally break the fine line of living matter in net-like arrangement between the epithelial and other bodies of the part.

When operations have peen performed in the manner described, and the fillings are as finely finished as suggested, they are the best for the preservation of remaining tissue protection of enamel over which the gold is placed, and for the prevention of the wedging of food against and the consequent recession of the gum; they subserve well the purpose of mastication, and present a beautiful appearance; in a word, the only way to keep permanently separate the margins of enamel of the proximate surfaces of the teeth and prevent recurrence of decay, is by full restoration of the contour of the tissue that is lost.

[TO BE CONTINUED.]

---

